# Geopolitical zones differentials in intermittent preventive treatment in pregnancy (IPTp) and long lasting insecticidal nets (LLIN) utilization in Nigeria

**DOI:** 10.1371/journal.pone.0254475

**Published:** 2021-07-16

**Authors:** Chinedu Chukwu, Herbert Onuoha, Kwala Adline Katty Okorafor, Oluwaseun Ojomo, Olugbenga A. Mokuolu, Michael Ekholuenetale

**Affiliations:** 1 Monitoring and Evaluation Unit, Management Sciences for Health, Abuja, Nigeria; 2 Department of Tropical Hygiene and Public Health, Medical Faculty, Heidelberg University, Heidelberg, Germany; 3 Department of Community Medicine, Faculty of Clinical Sciences, University of Abuja, Federal Capital Territory, Abuja, Nigeria; 4 Department of Psychology and Professional Development, Faculty of Health and Life Sciences, Oxford Brookes University, Oxford, United Kingdom; 5 Department of Paediatrics and Child Health, College of Health Sciences, University of Ilorin, Ilorin, Nigeria; 6 Department of Epidemiology and Medical Statistics, Faculty of Public Health, College of Medicine, University of Ibadan, Ibadan, Nigeria; Federal University of Agriculture, Abeokuta, NIGERIA

## Abstract

**Background:**

The coverage of long lasting insecticidal nets (LLIN) and intermittent preventive treatment of malaria in pregnancy (IPTp) uptake for the prevention of malaria commonly vary by geography. Many sub-Saharan Africa (SSA) countries, including Nigeria are adopting the use of LLIN and IPTp to fight malaria. Albeit, the coverage of these interventions to prevent malaria across geographical divisions have been understudied in many countries. In this study, we aimed to explore the differentials in LLIN and IPTp uptake across Nigerian geopolitical zones.

**Methods:**

We analyzed data from Nigeria Multiple Indicator Cluster Survey (MICS) 2016–17. The outcome variables were IPTp and LLIN uptake among women of childbearing age (15–49 years). A total sample of 24,344 women who had given birth were examined for IPTp use and 36,176 women for LLIN use. Percentages, Chi-square test and multivariable logit models plots were used to examine the geopolitical zones differentials in IPTp and LLIN utilization. Data was analyzed at 5% level of significance.

**Results:**

The overall prevalence of IPTp was 76.0% in Nigeria. Moreover, there were differences across geopolitical zones: North Central (71.3%), North East (76.9%), North West (78.2%), South East (76.1%), South South (79.7%) and South West (72.4%) respectively. Furthermore, the prevalence of LLIN was 87.7%% in Nigeria. Also, there were differences across geopolitical zones: North Central (89.1%), North East (91.8%), North West (90.0%), South East (77.3%), South South (81.1%) and South West (69.8%) respectively. Women who have access to media use, married, educated and non-poor were more likely to uptake IPTp. On the other hand, rural dwellers and those with media use were more likely to use LLIN. Conversely, married, educated, non-poor and women aged 25–34 and 35+ were less likely to use LLIN.

**Conclusion:**

Though the utilization of IPTp and LLIN was relatively high, full coverage are yet to be achieved. There was geopolitical zones differentials in the prevalence of IPTp and LLIN in Nigeria. Promoting the utilization of IPTp and LLINs across the six geopolitical zones through intensive health education and widespread mass media campaigns will help to achieve the full scale IPTp and LLIN utilization.

## Background

Approximately two hundred million malaria cases were recorded in African region, accounting for 92% of the global malaria cases [1, 2]. Nearly 50% of all the global cases of malaria recorded in 2017 were accounted for by five countries: four of which were in sub-Saharan Africa with Nigeria contributing a quarter (25%) of the total cases, followed by Democratic Republic of Congo with 11%, Mozambique contributing 5%, and Uganda with 4% [1]. Though there was a reduction of 20 million malaria cases as estimated in 2017, the 10 malaria most burdened countries in Africa still reported an upsurge of the disease. Among these 10 highest burdened countries, Nigeria and Madagascar as well as Democratic Republic of Congo had the highest increase with each accounting for more than 500,000 cases [1].

In Nigeria, malaria remains a major public health problem, taking its greatest toll on pregnant women and children under age 5. About 60% of all health facilities outpatients’ visits are due to malaria [3]. Which also accounts for 30% and 25% of under-5 and infant mortality respectively [3]. Moreover, about 11% of maternal mortality is traceable to malaria cases [3]. The enormous financial resources expended in the control and treatment of malaria shows the negative economic impact that it has on households. Also, malaria has caused a significant drawback to the socioeconomic growth of many households and high disability adjusted life years (DALYs). The concerted efforts of vector surveillance approach, the wide distribution of long lasting insecticide-treated nets (LLINs) as well as the educational campaigns should bring a reduction in malaria burden among the endemic regions. LLINs, among several other interventions, have significantly contributed to the reduction of the global burden of malaria since the year 2000. Over the years, as LLINs success is being evaluated, modification in the treatment of nets have emerged. These modifications were due to the insecticide-resistance by the malaria vectors and have led to the development of pyrethroid-synergist piperonyl butoxide (PBO) nets which is being given an interim endorsement by World Health Organization (WHO), as a class of vector control products [4–6].

The PBO LLIN as well as the non-pyrethroid indoor residual spraying interventions have shown an improvement in the control of malaria transmission, when compared with the standard LLINs in the regions with high prevalence of pyrethroid resistance. These have led the WHO to recommend that PBO LLIN be increased [4, 6]. The combination of indoor residual spraying (IRS) using pirimiphos-methyl and PBO-LLINs could not provide any added advantage in comparison with PBO-LLINs alone or the standard LLINs and IRS. Though LLINs have been widely used by most people at risk of malaria, a great deal of communities is yet to maximize the available malaria control interventions in order to control the disease. There are ample evidence that some people in the malaria endemic regions do not have access to LLINs and therefore do not use it at all [5].

Malaria being one of the prominent causes of morbidity and mortality in resource-constrained settings, brought about the Roll Back Malaria Initiative (RBMI) which identified the use of LLIN as a key strategy for malaria prevention especially for women and under-5 children [7]. To minimize the socioeconomic impact that malaria brought in Nigeria, the Federal Ministry of Health and Roll Back Malaria partners adopted the Scale-Up for Impact (SUFI) approach enshrined in the revised edition of the National Malaria Strategic Plan (NMSP). The target of this approach was to increase the ownership of LLINs and its use among the key population [8]. The control of malaria vector has provided a preventive line of attack to the incidence of complications and deaths caused by the disease. Interestingly, the WHO is also using the combine duo approaches of IRS and LLINs to control malaria [9].

Furthermore, WHO has recommended the use of IPTp for malaria prevention during pregnancy [10]. The efforts to scale-up IPTp using Sulfadoxine-Pyrimethamine (SP) amongst others have been slow in some African countries. To aid this process, health workers have asked several questions around the administration and use of IPTp-SP especially that it must be taken as directly observed therapy. It was against this backdrop that WHO is expanding on her recommendations to national health authorities to publicize these recommendations to their citizens and enforce their correct application [10]. One of these recommendations is to protect pregnant women with SP antimalarial drug in regions where there are moderate and high transmission of malaria [11]. In Nigeria, only 26.2% of pregnant women took three doses or more of IPTp as recommended by the WHO [12].

Previous studies have identified geographical divisions as a key predictor in the uptake of LLIN, IPTp as well as other maternal health care interventions. For example, regional differences emerged as the most important predictor of insecticide-treated nets (ITN) ownership and use in Nigeria, according to a subnational profiling analysis [13]. In Tanzania, women’s geographical zones of residence was a significant predictor of IPTp uptake [14, 15]. In another previous study, being a resident of the Eastern Zone was linked to increased IPTp uptake when compared with the Lake Zone [14]. In Mali, geographical region was associated with IPTp uptake [16]. Another study reported that women from southern Mozambique were more likely to uptake IPTp when compared with those from the northern region [17].

In Nigeria, there is large peculiarity in health care-seeking behaviours across geographical regions. For example, women from the southern region comprising of south-south, south-east and south-west are believed to be more educated than their counterpart in the northern region (north-central, north-east and north-west) and therefore could be more likely to uptake health care services. Women’s education or enlightenment has been reported to be positively associated with health care services uptake [18–22]. Other geographical region-based factors such as ethnicity, cultural norms, religion amongst others could influence the uptake of IPTp and LLIN as practiced in the region. Despite the fact that huge distribution efforts regarding malaria prevention and control have been going on in Nigeria since over a decade, to the best of our knowledge, studies that examined the rate of consumption of such commodities, especially the regional patterns, are lacking in literature. In light of the above, this study aimed to examine the differentials in IPTp and LLINs uptake among women of reproductive age across the six geopolitical zones in Nigeria.

## Methods

### Data sources

We utilized a nationally representative large data from Nigeria Multiple Indicator Cluster Survey (MICS) 2016–17. A total sample of 24,344 women who had given birth were included for IPTp use and 36,176 women for LLIN use were included for analysis. Through this survey, estimates for a large quantity of indicators concerning the condition of reproductive age women at both rural and urban areas of the 6 geopolitical zones of Nigeria was made. The main sampling strata in the survey were the states in each geopolitical zone, while the primary sampling units (PSUs) were identified as the Enumeration Areas (EAs). The National Integrated Survey of Households round 2 (NISH2) master sample which was developed for the most recent population census, served as the source of EAs for the survey. They employed two-stage sampling: (1) selection of EAs and (2) selection of households. The data can be freely accessed for research purposes on: https://mics.unicef.org/surveys. From the URL, select country (Nigeria), select the survey year (2016–17) and download the data.

### Data collection instrument

The MICS 2016–2017 utilized four distinct questionnaires. The first is the household questionnaire, which was used for the collection of household characteristics and basic sociodemographic information of all the household members; the second questionnaire was the individual women questionnaire, designed to collect information from all the women of reproductive age (15–49 years), present in each household; the third questionnaire was the individual men questionnaire. This was designed to elicit information from all men in every other household (one man in every two households) who are within the age of 15 and 49 years; and the fourth questionnaire was for children who were less than 60 months (under-five year children). This fourth questionnaire was administered to the mothers of the children or their caregivers who also live in the sampled households. The following modules were included in the individual women questionnaire: the information panel of the woman, her background, her accessibility to mass media as well as the utilization of information/communication technology, her fertility/birth history, what her desire for last birth was, the maternal and newborn health, the post-natal healthcare checks, any illness symptoms, utilization of contraceptive methods and any unmet need for contraception, any female genital mutilation/cutting, her attitude towards domestic violence/intimate partner violence, her marriage/union status, sexual behaviour, sexually transmitted infection, tobacco and alcohol use and life satisfaction.

### Definition of variables

#### Outcome variable

Our outcome variables were IPTp and LLIN. These were measured dichotomously as yes vs. no if a woman used or did not use.

#### Explanatory variables

The explanatory variables included in this study were selected based on previous studies [13–17, 23], and are presented in Table 1 below.

**Table 1:** Categories and operational definition of independent variables.

	Variables	Definitions/categories
1	Age (in years)	Age of the respondent (<25, 25–34, 35+)
2	Geopolitical zone	This is the region of residence for a respondent (North Central, North East, North West, South East, South South, South West)
3	Residential status	This is the area of residence of respondents (urban, rural)
4	Wealth quintiles*	Economic/wealth status of the household (poor, non-poor)
5	Marital status	Unmarried (singles and formerly married) and married
6	Exposure to media	This was generated from whether a respondent used any of newspaper/magazine, radio or television (yes, no)
7	Education	Respondent’s formal level of education/schooling (such as; no formal education, primary level, secondary/higher level
8	Number of children ever born	Total number of children ever born (nil, <4, 4+)
9	Ethnicity	Hausa, Igbo, Yoruba, Other ethnic groups
10	State	North Central: 1- Benue, 2- Kogi, 3- Kwara, 4- Nasarawa, 5- Niger, 6- Plateau, 7- Federal Capital Territory
		North East: 1- Adamawa, 2- Bauchi, 3- Borno, 4- Gombe, 5- Taraba, 6- Yobe
		North West: 1- Jigawa, 2- Kaduna, 3- Kano, 4- Katsina, 5- Kebbi, 6- Sokoto, 7- Zamfara
		South East: 1- Abia, 2- Anambra, 3- Ebonyi, 4- Enugu, 5- Imo
		South South: 1- Akwa-Ibom, 2- Bayelsa, 3- Cross River, 4- Delta, 5- Edo, 6- Rivers
		South West: 1- Ekiti, 2- Lagos, 3- Ogun, 4- Ondo, 5- Osun, 6- Oyo

* For the calculation of household wealth status, household assets such as ownership of electronics and means of transportation (example television and bicycle), house building material quality (example floor, wall and roof types) were considered. Principal component analysis was used to generate factor scores which were assigned to each item and these scores were summed and standardized for each household. The standardized household scores placed each household in a continuous scale according to their relative wealth scores. These scores were subsequently categorized in binary form to rank the households into poor and non-poor households [24].

The MICS data had already developed and classified household wealth quintile as a variable into five groups, each of which was worth 20% of the total: poorest, poorer, middle, richer, and richest. We re-categorized household wealth quintiles into two categories for the analysis: poor (poorest, poorer) and non-poor (middle, richer and richest) [25, 26].

### Ethical consideration

The data used in this study is publicly available and the authors of this manuscript were not involved in the collection of data from the participants. The authors sought for permission from MICS and access to the data was granted after the request was considered and approved. MICS Program is consistent with the standards for ensuring the protection of respondents’ privacy. No further approval was required for this study since MICS Programs are in consistent with the standards for ensuring the protection of respondents’ privacy.

### Analytical approach

To compute the estimates, we adjusted for sampling weights, stratification and clustering by using the survey (‘svy’) module. At the univariate level, the frequency distribution of relevant variables was calculated, the chi-square distribution test of association was calculated at the bivariate level, and the logit model plot was created to determine the geopolitical zones differences in IPTp and LLIN utilization in Nigeria. This approach is consistent with previous studies [27, 28]. Variables that were not statistically significant at the bivariate level were not included in the adjusted logit model plot.

The logistic regression model was of the form:

$$\text{log}\;\left(\text{p}/\left(1-\text{p}\right)\right)=\beta_{0}+\beta_{1}{\text{x}}_{1}+\beta_{2}{\text{x}}_{2}+\cdots \beta_{\text{m}}{\text{x}}_{\text{m}};$$

where p indicates the probability of IPTp uptake or LLIN use and β_is_ are the regression coefficients associated with the reference group and the x_i_ are the explanatory variables. The p-value of <0.05 was set as being statistically significant. We analyzed the data using the version 14 of Stata statistical software from StataCorp., College Station, Texas, United States of America.

## Results

Table 2 showed the pooled prevalence and differentials by geopolitical zones of IPTp in Nigeria. The overall prevalence of IPTp was 76.0% in Nigeria. Moreover, there were differences across geopolitical zones. The South South zone had the highest prevalence of IPTp (79.7%), followed by North West with 78.2%, North East (76.9%), South East (76.1%), South West (72.4%) and the least prevalence is the North Central zone with a prevalence of (71.3%). In addition, the prevalence were examined across respondents’ characteristics whereby the results showed within respondents characteristics and between geopolitical zones differentials respectively. The prevalence of IPTp was statistically different by women’s exposure to media, marital status, education, ethnicity and state of origin in north-central region (p<0.05). Furthermore, the prevalence of IPTp was statistically different by women’s media use, wealth status and state of origin in north-east region (p<0.05). In the north-west, IPTp prevalence was statistically different by women’s age, exposure to media use, residential status, wealth status and state of origin (p<0.05). IPTp prevalence was statistically different by women’s exposure to media, education, wealth status and the state of origin in south-east region (p<0.05). In addition, IPTp prevalence was statistically different by exposure to media, marital status, education, wealth status and state of origin in south-south region (p<0.05). In south-west region, the prevalence of IPTp was statistically different by women’s state of origin (p<0.05). Chi-square test was used to determine the statistical significance. Table 2 showed more details on this.

**Table 2:** Distribution of intermittent preventive treatment in pregnancy in Nigeria.

Variable	n (%)	North Central (n = 4,808)	North East (n = 3,922)	North West (n = 7,341)	South East (n = 2,124)	South South (n = 3,109)	South West (n = 3,040)	Pooled sample (n = 24,344)
**Age**								
<25	4,374 (18.0)	72.0	75.7	76.4	73.9	78.3	72.9	75.2
25–34	9,800 (40.3)	71.0	77.5	80.9	76.6	81.0	72.4	76.8
35+	10,170 (41.8)	71.3	77.7	75.4	76.6	77.6	72.1	75.0
p-value		0.953	0.762	0.019*	0.779	0.474	0.981	0.239
**Media use**								
Yes	15,138 (62.2)	74.4	80.3	80.8	78.3	81.7	73.2	78.1
No	9,206 (37.8)	64.9	74.0	74.1	67.6	63.1	65.4	71.0
p-value		<0.001*	0.012*	<0.001*	0.004*	<0.001*	0.092	<0.001*
**Number of children born**								
<4	11,330 (46.5)	72.0	76.7	77.7	76.3	81.3	72.6	76.0
4+	13,014 (53.5)	70.5	77.2	78.5	75.7	77.3	72.0	75.9
p-value		0.566	0.839	0.660	0.843	0.119	0.843	0.896
**Marital status**								
Unmarried	1,907 (7.8)	58.7	75.0	79.2	81.0	72.5	73.1	73.4
Married	22,397 (92.2)	72.0	77.0	78.2	75.6	80.6	72.3	76.1
p-value		0.049*	0.745	0.866	0.354	0.038*	0.907	0.226
**Residential status**								
Urban	6,964 (28.6)	71.9	80.5	84.0	80.5	83.1	71.9	77.4
Rural	17,380 (71.4)	71.1	75.9	76.1	74.7	78.3	73.9	75.2
p-value		0.742	0.129	<0.001*	0.100	0.086	0.531	0.033*
**Education**								
None-non-formal	10,525 (43.2)	67.2	74.8	77.3	59.3	52.2	67.7	74.5
Primary	4,442 (18.2)	68.0	79.5	75.9	71.3	75.5	74.3	73.9
Secondary	7,183 (29.5)	74.5	78.5	80.7	74.9	80.8	72.3	76.8
Higher	2,194 (9.0)	77.1	82.4	85.4	88.6	85.2	73.2	80.7
p-value		0.023*	0.273	0.076	0.001*	0.001*	0.714	<0.001*
**Ethnicity**								
Hausa	10,554 (43.4)	74.8	79.5	78.5	80.0	71.4	88.9	78.4
Igbo	2,836 (11.6)	73.8	71.4	88.0	75.7	84.0	71.1	76.1
Yoruba	2,958 (12.2)	56.2	75.0	87.5	100.0	86.4	72.4	70.8
Other ethnic group	7,996 (32.8)	72.4	72.5	70.6	92.3	79.1	70.1	74.9
p-value		<0.001*	0.062	0.074	0.455	0.535	0.252	<0.001*
**Wealth status**								
Poor	9,952 (40.9)	69.1	73.8	74.5	52.1	68.8	70.9	72.3
Non-poor	14,392 (59.1)	72.3	80.0	82.1	79.2	80.6	72.5	77.6
p-value		0.248	0.013*	<0.001*	<0.001*	0.012*	0.751	<0.001*
**State**								
1		58.5	74.0	75.7	85.3	81.0	48.3	
2		75.3	79.9	78.3	78.6	73.1	76.4	
3		50.4	72.3	77.1	63.9	85.4	78.1	
4		85.5	77.6	80.8	78.4	72.5	85.3	
5		70.7	79.2	78.8	74.0	81.5	57.5	
6		87.0	77.3	68.3		85.7	75.6	
7		62.5		89.2				
p-value		<0.001*	<0.001*	<0.001*	0.011*	<0.001*	<0.001*	
**Total estimate**	**24,344**	**71.3**	**76.9**	**78.2**	**76.1**	**79.7**	**72.4**	**76.0**

North Central: 1- Benue (n = 603), 2- Kogi (546), 3- Kwara (n = 505), 4- Nasarawa (n = 769), 5- Niger (n = 868), 6- Plateau (n = 753), 7- Federal Capital Territory (n = 764)

North East: 1- Adamawa (n = 688), 2- Bauchi (n = 935), 3- Borno (n = 301), 4- Gombe (n = 735), 5- Taraba (n = 559), 6- Yobe (n = 704)

North West: 1- Jigawa (n = 900), 2- Kaduna (n = 810), 3- Kano (n = 1,946), 4- Katsina (n = 964), 5- Kebbi (n = 846), 6- Sokoto (n = 883), 7- Zamfara (n = 992)

South East: 1- Abia (n = 420), 2- Anambra (n = 447), 3- Ebonyi (n = 454), 4- Enugu (n = 370), 5- Imo (n = 433)

South South: 1- Akwa-Ibom (n = 674), 2- Bayelsa (n = 540), 3- Cross River (n = 528), 4- Delta (470), 5- Edo (n = 464), 6- Rivers (n = 433)

South West: 1- Ekiti (n = 355), 2- Lagos (n = 985), 3- Ogun (n = 430), 4- Ondo (n = 468), 5- Osun (n = 354), 6- Oyo (n = 448)

*Significant at p<0.05

*P* was obtained using Chi-square test

Results from Table 3 showed the pooled prevalence and differentials by geopolitical zones of LLINs utilization among women of reproductive age in Nigeria. The overall prevalence of LLIN was 87.7%% in Nigeria. Furthermore, there were changes across geopolitical zones. The North East zone had the highest prevalence of LLINs (91.8%), followed by North West with 90.0%, North Central (89.1%), South South (81.1%), South East (77.3%) and the least prevalence is the South West zone with a prevalence of (69.8%). In addition, the prevalence were examined across respondents’ characteristics whereby the results showed within respondents characteristics and between geopolitical zones differentials respectively. The prevalence of LLIN was statistically different by women’s age, residential status, ethnicity and state of origin in north-central region (p<0.05). Furthermore, the prevalence of LLIN was statistically different by women’s age, marital status and state of origin in north-east region (p<0.05). LLIN prevalence was statistically different by women’s exposure to media, marital status, ethnicity and state of origin in north-west region (p<0.05). In addition, LLIN prevalence was statistically different by women’s age, number of children ever born, marital status, education, wealth status and state of origin in south-east region (p<0.05). In south-south region, the prevalence of LLIN was statistically different by women’s age, media use, marital status, residential status, wealth status and state of origin (p<0.05). The prevalence of LLIN was statistically different by women’s age, number of children ever born, marital status and state of origin in south west (p<0.05). The statistical significance was determined using chi-square test. Table 3 showed more details.

**Table 3:** Distribution of long lasting insecticidal nets utilization among women of reproductive age in Nigeria.

Variable	n (%)	North Central (n = 7,462)	North East (n = 5,469)	North West (n = 9,765)	South East (n = 3,753)	South South (n = 4,918)	South West (n = 4,809)	Pooled sample (n = 36,176)
**Age**								
<25	12,526 (36.4)	91.7	94.4	91.6	83.2	86.7	80.0	90.8
25–34	11,229 (32.7)	90.0	91.0	89.7	71.2	76.4	60.4	86.8
35+	10,621 (30.9)	87.1	90.5	89.6	75.6	78.9	69.1	86.3
p-value		0.023*	0.011*	0.116	0.013*	0.003*	0.001*	<0.001*
**Media use**								
Yes	22,755 (62.9)	88.0	91.4	91.7	75.8	79.8	68.1	86.5
No	13,421 (37.1)	90.4	92.0	88.4	81.4	86.4	76.9	89.2
p-value		0.074	0.549	<0.001*	0.148	0.046*	0.076	<0.001*
**Number of children ever born**								
Nil	10,032 (29.2)	92.2	93.1	92.2	82.8	85.9	80.2	90.1
1–3	11,330 (33.0)	88.3	91.6	89.1	71.6	78.8	66.5	86.3
4+	13,014 (37.9)	89.2	91.7	90.3	75.0	78.9	66.1	88.2
p-value		0.072	0.627	0.065	0.017*	0.050	0.017*	<0.001*
**Marital status**								
Unmarried	10,411 (30.4)	91.4	94.8	94.7	81.6	86.7	78.2	90.2
Married	23,891 (69.6)	89.0	91.4	89.5	73.5	77.1	66.0	87.4
p-value		0.129	0.022*	<0.001*	0.018*	<0.001*	0.008*	<0.001*
**Residential status**								
Urban	11,689 (32.3)	83.0	93.7	91.3	74.8	72.9	68.2	84.8
Rural	24,487 (67.7)	90.4	91.3	89.7	77.9	82.7	73.1	88.6
p-value		<0.001*	0.112	0.130	0.459	0.005*	0.241	<0.001*
**Education**								
None-non-formal	12,055 (35.1)	89.8	92.4	90.2	90.2	93.6	75.4	90.6
Primary	5,213 (15.2)	90.6	90.9	90.8	83.6	81.1	69.1	87.5
Secondary	13,452 (39.1)	89.7	92.2	90.0	76.9	80.7	68.4	85.4
Higher	3,656 (10.6)	86.3	88.8	92.9	63.3	77.1	69.4	81.4
p-value		0.464	0.530	0.729	0.001*	0.248	0.774	<0.001*
**Ethnicity**								
Hausa	14,073 (38.9)	86.2	92.6	90.5	0.0	100.0	85.7	90.7
Igbo	4,957 (13.7)	69.7	85.7	91.7	77.5	76.9	71.4	77.3
Yoruba	4,702 (13.0)	79.1	100.0	92.9	50.0	85.7	69.2	72.0
Other ethnic group	12,444 (34.4)	90.8	90.0	83.5	66.7	81.3	71.7	87.4
p-value		<0.001*	0.134	0.001*	0.216	0.779	0.781	<0.001*
**Wealth status**								
Poor	14,301 (39.5)	89.7	91.8	90.0	87.5	88.2	77.0	90.0
Non-poor	21,875 (60.5)	88.5	91.7	90.2	73.9	79.5	68.0	85.2
p-value		0.400	0.973	0.789	0.001*	0.008*	0.062	<0.001*
**State**								
1		89.2	89.2	93.9	62.4	73.3	54.1	
2		92.7	87.8	86.1	72.9	89.1	73.3	
3		88.2	95.3	92.3	87.2	84.0	83.8	
4		90.5	94.9	88.5	83.1	80.0	81.4	
5		81.6	88.1	81.9	47.9	78.1	60.0	
6		93.4	93.9	85.7		80.5	63.4	
7		83.6		93.6				
p-value		<0.001*	<0.001*	<0.001*	<0.001*	<0.001*	<0.001*	
**Total estimate**	**36,176**	**89.1**	**91.8**	**90.0**	**77.3**	**81.1**	**69.8**	**87.7**

North Central: 1- Benue (n = 946), 2- Kogi (n = 960), 3- Kwara (n = 747), 4- Nasarawa (n = 1,155), 5- Niger (n = 1,225), 6- Plateau (n = 1,172), 7- Federal Capital Territory (n = 1,257)

North East: 1- Adamawa (n = 1,007), 2- Bauchi (n = 1,193), 3- Borno (n = 425), 4- Gombe (n = 1,030), 5- Taraba (n = 881), 6- Yobe (n = 933)

North West: 1- Jigawa (n = 1,176), 2- Kaduna (n = 1,247), 3- Kano (n = 2,576), 4- Katsina (n = 1,192), 5- Kebbi (n = 1,176), 6- Sokoto (n = 1,142), 7- Zamfara (n = 1,256)

South East: 1- Abia (n = 700), 2- Anambra (n = 812), 3- Ebonyi (n = 781), 4- Enugu (n = 744), 5- Imo (n = 716)

South South: 1- Akwa-Ibom (n = 1,026), 2- Bayelsa (n = 816), 3- Cross River (n = 802), 4- Delta (n = 731), 5- Edo (n = 775), 6- Rivers (n = 768)

South West: 1- Ekiti (n = 552), 2- Lagos (n = 1,584), 3- Ogun (n = 678), 4- Ondo (n = 657), 5- Osun (n = 630), 6- Oyo (n = 708)

*Significant at p<0.05

*P* was obtained using Chi-square test

**Fig 1:**
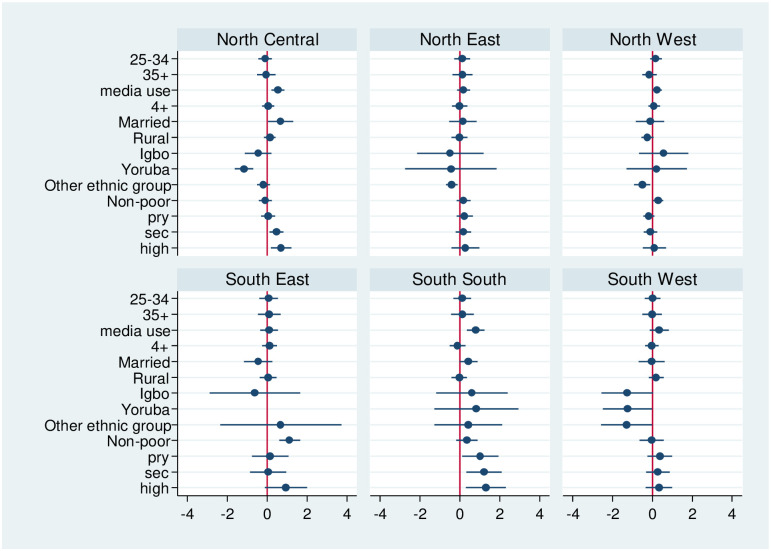
Estimates of the odds of IPTp by respondents’ characteristics.

Notably, only the variables which were statistically significant at the bivariate level (p<0.05) that were included. Results in Fig 1 showed that women who have access to media use, married and educated were more likely to uptake IPTp in North Central and South South, when compared with women without media use, unmarried and with no formal education respectively. Non-poor women from South East were more likely to uptake IPTp.

**Fig 2:**
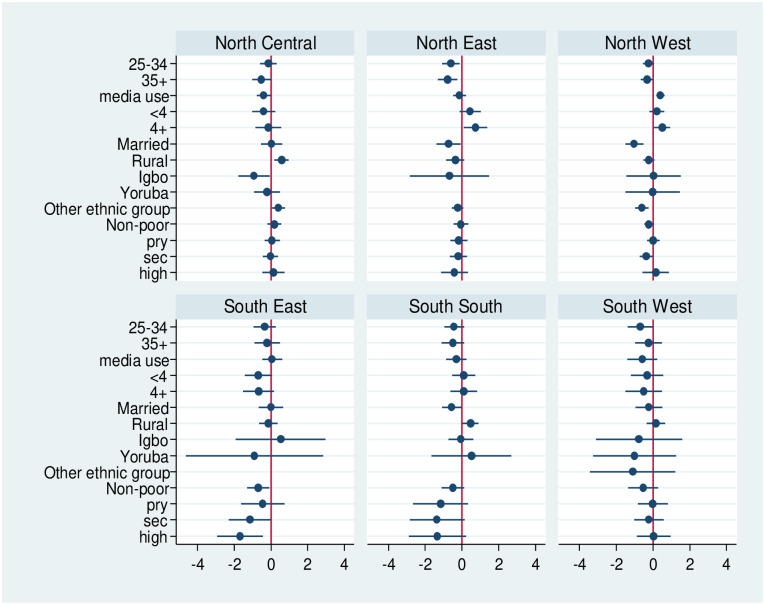
Estimates of the odds of long lasting insecticidal nets utilization respondents’ characteristics.

In the bivariate analysis using chi-square test, only the variables which were statistically significant (p<0.05) were included. Fig 2 showed that rural dwellers were more likely to use LLINs, when compared with urban dwellers in North Central and South South respectively. Married women were less likely to use LLINs, when compared with unmarried women in North West. In addition, educated women were less likely to use LLINs, when compared with women with no formal education in South Eastern and South Southern States of Nigeria. In the North East, women aged 25–34 and 35+ were less likely to utilize long lasting insecticidal nets.

## Discussion

This study has become one of the foremost to explore geopolitical zones differentials in IPTp and LLINs utilization in Nigeria. Our findings showed that there are disparities in IPTp uptake by geopolitical zones. This is consistent with the report of a previous study [29]. There were differences in IPTp uptake by geopolitical zones with North Central having the least coverage in Nigeria. Similarly, differences in LLINs utilization was identified. While South West had the least utilization, North East had the highest utilization respectively. Though massive campaigns for the distribution of LLINs have been going on for over a decade, only few studies have attempted to assess the rate of LLIN use. However, the studies did not report the rate of LLINs utilization before or after the massive campaigns, in both Nigeria and other sub-Saharan African countries [30–32]. Several factors dependent on geography, such as ethnicity, cultural norms and women’s enlightenment could have influenced IPTp and LLIN utilization in Nigeria.

The findings from this study revealed the factors associated with LLINs utilization and these varied by geopolitical zones. Rural dwellers, women who ever given birth to at least four children and those who use media were more likely to utilize LLINs, when compared with urban residents, those who had ever given birth to less than four children and who do not use the media. Conversely, the married women, educated, non-poor and those of advance age were less likely utilize LLINs when compared with the unmarried, uneducated, poor, and those below 25 years. This is in line with the findings of a previous study [23], where the rural dwellers had an increased LLINs utilization when compared with their counterparts from urban residence [23]. Another study that assessed the impact mass media campaign had on bed net utilization in Ethiopia showed that mass media has great influence in communicating to the people the need for LLINs and encouraging them to use it [33]. Contrary to our results, a previous study reported the use of LLINs in Eastern Nigeria was higher among small size families (family size less than four), compared to larger families [7]. Though the effectiveness of LLINs in protecting against malaria is evident, it is still difficult to achieve a corresponding ownership and utilization among women, particularly, the vulnerable or most at-risk population. This can be due to several factors and we recommend that future researchers could examine the cultural norms, perception, acceptability, myths and misconceptions using qualitative research approach.

Furthermore, the findings of this study showed that women who use media, married, educated and non-poor were more likely to uptake IPTp, when compared with their counterpart without media use, unmarried, with no formal education and poor respectively. As expected, married women are more eligible for the uptake of IPTp and the finding of higher uptake among the married folks is not a surprise. Similarly, the findings showed that educated women were more likely to uptake IPTp, when compared with those with no formal education. This agrees with the notion that educated women would have more knowledge about the benefits of malaria prevention during pregnancy. Women who were exposed to media use were more likely to have IPTp uptake, when compared to their counterpart who were not exposed to media use. Education and exposure to media use would enhance accessibility to behaviour change communication and consequently lead to increased health care utilization. The non-poor women were more likely to have IPTp uptake. It is assumed that the non-poor women are able to afford out-of-pocket expenditures for health care services, especially in resource-constrained settings [34–38]. Also, improved socioeconomic status among women is a predetermining factor for IPTp utilization.

### Strengths and limitations

This is one of the foremost studies to be conducted on a nationally representative sample of adult women in Nigeria, to investigate geopolitical zones differentials in the utilization of IPTp and LLIN. As the study is based on a secondary dataset, the researcher has limited control over the type of data elements available for analysis. Though Nigeria is currently one of the leading malaria high burdened countries, not much progress is presently being made to reduce the malaria burden. Information on the use LLINs was based on self-report by participants. Based on the data collection methodology, data collectors observed the availability of LLIN in each household to ascertain whether they were hung or not hung, and whether they were kept in their original packaging. Moreover, the participants were asked who slept under net the night preceding the survey, by the data collector. As such, information bias may have occurred due to self-reporting, thereby posing a possible limitation. The actual use of LLINs by the participant the previous night before the survey was not verified.

## Conclusion

This study shows that the utilization of IPTp and LLIN was relatively high despite that full coverage were yet to be achieved. Specifically, coverage was significantly different across geopolitical zones. Promoting the utilization of IPTp and LLINs by the most-at-risk across the six geopolitical zones through intensified health education and mass media campaigns will enhance full scale IPTp and LLIN utilization in Nigeria. There is need to enhance the strategies to increase IPTp and LLINs utilization as there are shortfalls in the national targets The findings of this study should be used by stakeholders who are involved in the massive distribution of LLINs and promotion of IPTp with emphasis on behavioural change communication especially among the pregnant women.

## Abbreviations

DALY: Disability adjusted-life-years
EA: Enumeration Area
IPTp: Intermittent preventive treatment of malaria in pregnancy
IRS: Indoor residual spraying
ITN: Insecticide-treated nets
LLIN: long lasting insecticidal nets
MICS: Multiple Indicator Cluster Survey
NISH: National Integrated Survey of Household
NMSP: National Malaria Strategic Plan
PBO: Piperonyl butoxide
PSU: Primary Sampling Unit
RBMI: Roll Back Malaria Initiative
SP: Sulfadoxine-Pyrimethamine
SSA: Sub-Saharan Africa
SUFI: Scale-Up for Impact
