# Correction: Geopolitical zones differentials in intermittent preventive treatment in pregnancy (IPTp) and long lasting insecticidal nets (LLIN) utilization in Nigeria

**DOI:** 10.1371/journal.pone.0260209

**Published:** 2021-11-12

**Authors:** The PLOS ONE Staff

There is an error in the special author designations; the publisher apologizes for the error. The correct designation is: ‡ ME and CC are joint first authors on this work.
